# An explainable multi-backbone deep feature fusion framework for intelligent surveillance and citizen safety in smart city ecosystems

**DOI:** 10.3389/frai.2026.1836157

**Published:** 2026-05-29

**Authors:** Tamara Zhukabayeva, Zulfiqar Ahmad, Dina Satybaldina, Didar Yedilkhan

**Affiliations:** 1 Faculty of Information Technology, L.N. Gumilyov Eurasian National University, Astana, Kazakhstan; 2 Department of Computer Engineering, Astana IT University, Astana, Kazakhstan; 3 Department of Computer Science and Information Technology, Hazara University, Mansehra, Pakistan; 4 Scientific Research Institute of Information Security and Cryptology, L.N. Gumilyov Eurasian National University, Astana, Kazakhstan

**Keywords:** deep learning, ensemble learning, explainable AI, grad-CAM, LIME, smart city surveillance, violence detection

## Abstract

**Introduction:**

Safety in the city has become a pressing issue in modern smart cities because of the rising rate of violence, attacks, and incidences involving weapons recorded by extensive CCTV networks. Conventional surveillance systems also have difficulties in real-time recognition, scaling, and transparency, as they are not always able to identify cases of violence on time.

**Methods:**

This paper proposes DeReV, a novel explainable deep feature-level ensemble framework that integrates two variants from each of the DenseNet, ResNet, and VGG families within a multi-backbone feature fusion architecture for multi-class violence detection in videos. The model is based on the Smart-City CCTV Violence Detection (SCVD) dataset to classify CCTV video as either normal, violent, or weaponized.

**Results and discussion:**

DeReV resulted in better performance with an accuracy of 96.15 and a macro F1-score of 0.9617, which is better than the individual baseline networks such as ResNet101 (91.67%), DenseNet169 (92.30%), and VGG16 (92.94%). The ROC analysis has shown that the AUC scores are 0.99 (normal), 0.98 (violence), and 0.98 (weaponized), whereas the Precision-Recall curves are 0.99, 0.97, and 0.95, respectively, which prove the high capability of discrimination and stability under different conditions. The interpretability and transparency of the results were achieved by using Explainable AI (XAI) methods like Grad-CAM, LIME, and Counterfactual Temporal Importance to identify salient spatial and temporal features in the predictive behaviors of the decision outcomes. The suggested DeReV framework not only offers more accurate predictions and model explainability but also supports trustworthy near real-time and citizen-centered AI surveillance, building safer and more resilient smart city ecosystems.

## 1 Introduction

The concept of a smart city ecosystem has become one of the foundations of sustainable development in urban areas where complex technologies are combined to increase the efficiency of operations, environmental sustainability, and overall quality of life of the citizens (Wen et al., 2023; Afzal et al., 2025; Alwakid et al., 2025). As cities continue their explosive population expansion and city infrastructures become more complex and demand more optimization, municipalities are embracing intelligent systems that embrace artificial intelligence (AI), the Internet of Things (IoT), and data analytics. Such ecosystems make cities more responsive, flexible, and reactive to dynamic threats, including but not limited to public safety, traffic control, energy use, and environmental surveillance. This area is one of the important ones, as the security and safe conditions become important pillars of smart cities’ development because a secure and peaceful environment leads to social stability and city sustainability. The use of AI-based video analytics to develop intelligent surveillance technologies is becoming more popular in the monitoring of the general population, the identification of suspicious behavior, and real-time support of decisions by law enforcement in response to this behavior. These systems ensure not only the quickness and the precision of the incident identification but also minimize the use of the manual surveillance, thus ensuring proactive crime prevention and the improved level of the safety and trust of the city inhabitants (Ramírez-Moreno et al., 2021; Li et al., 2021; Sharma and Kanwal, 2024; Safi et al., 2022).

The use of intelligent surveillance systems is important in improving the safety of citizens, traffic control, and intensifying crime control in smart cities. These systems will enable visual data surveillance and analysis to take place everywhere in the city, such as streets, transport stations, and neighborhoods, by taking into consideration high-resolution computer vision, deep learning, and real-time data analysis. In terms of the security of citizens, AI-based surveillance can automatically detect abnormal or violent behaviors, unattended objects, or weapon-like activities to enable the authorities to make timely and effective intervention before the issue becomes unmanageable. Intelligent video analytics are applied to support traffic management to help identify vehicles, congestion, and violation maintenance, which result in easier mobility, fewer accidents, and emergency response (Huang et al., 2022; Myagmar-Ochir and Kim, 2023; Akintayo et al., 2025). These systems also facilitate crime prevention as they apply predictive analytics to identify high-risk areas and typical behavioral patterns that can help the law enforcement agencies to change to proactive policing methods. Intelligent surveillance is capable of enhancing situational awareness and operational efficiency, as well as establishing the foundation of safer, resilient, and sustainable urban populations, which may be directly traced to the objectives of a smart city in general (Wu et al., 2021; Biswas and Wang, 2023).

Smart cities have the opportunity to capture and react to suspicious or violent acts automatically with the integration of AI and computer vision into the publicly used surveillance systems. In the conventional approach to surveillance, the manual aspect of surveillance is mostly employed, and this is time-consuming as well as subject to human error, particularly in dealing with the large-scale networks of cameras. In comparison, AI-based video analytics can be used to deliver automatic interpretation of visual information, enabling the recognition of complicated behavioral patterns and possible threats in real time. Computer vision systems, especially the deep learning-based models like the convolutional neural networks (CNNs) architecture, can effectively detect abnormal movements, gestures, and interactions (Sadri et al., 2021; Valipoor and de Antonio, 2023; Hussain et al., 2024). This feature will enable the early identification of any situation that might include fights, attacks, or weapons appearing in open areas. Moreover, with the continuous learning systems, such models can be tuned to support different environments, lighting conditions, and the density of crowds, which will guarantee robustness and reliability in a variety of urban conditions. The combination of AI and computer vision will thereby change the public surveillance system to an active and proactive form of safety mechanism rather than a passive instrument of record keeping, able to aid law enforcement and enhance the security of citizens in real time (Soliman et al., 2023; Bin Islam et al., 2023; AlHaddad et al., 2023; Tumati and Arepalli, 2025).

The situation in urban safety is one of the most urgent topics of contemporary urban society, where high population density and mobility contribute to the risk of violence, attacks, and violence with the usage of weapons. Transport terminals, educational and shopping facilities, and entertainment facilities are the most susceptible to spontaneous physical aggression or escalations of conflict. Physical fighting, armed robberies, knives and gun attacks are only a few of the incidents that not only threaten lives but also cause social panic and economic turmoil on a large scale. Conventional security systems such as manual CCTV surveillance and responsive law enforcement action tend to offer in-time responses because human factors limit processing of huge amounts of visual evidence (Li et al., 2021; Zahra et al., 2021; Hu Q. et al., 2025; Gu et al., 2025). Due to this, numerous incidents of violence or use of weapons are not reported in time before a lot of damage has been inflicted. The increasing rate of such events highlights the ultimate importance of preliminary and efficient detection systems that can be used to detect the possible threat before it turns into a severe event. Smart video surveillance using AI and deep learning is a radical solution that will allow real-time detection, classification, and alerts of suspicious behavior and promote proactive prevention and will allow making the city a safer place (Alwakid et al., 2025; Luo et al., 2023; Hu F. et al., 2025).

Although there has been enormous progress in computer vision and deep learning, there are several major constraints in the current surveillance and violence detection models that prevent their application in real-world smart city settings. The existing methods are mostly based on the single convolutional neural network (CNN) models like VGG or ResNet, which, despite being effective in terms of their capabilities to classify the still image, tend to be less efficient in terms of their ability to generalize over a wide range of possible scenarios associated with urban environments (different light conditions, camera views, crowd sizes, and movement) (Tumati and Arepalli, 2025; Mascarenhas and Agarwal, 2021; Faghihi et al., 2023; Kausar et al., 2021). Moreover, such models are usually encapsulated, meaning they are not very interpretable or transparent in how they make their decision. The other significant issue to face is the fact that the traditional models capture very limited temporal and fine differences between normal, violent, and weaponized behaviors. Violence and aggression are strongly temporal, with the order and intensity of actions having a deep meaning, but most models pay much attention to spatial characteristics, not to motion and time context. Also, the issue of achieving real-time performance is a long-standing bottleneck, with the cost of deep learning inference being high and conflicting with the requirements of smart city surveillance systems in terms of both speed and scalability. All these issues would point to the need for an intelligent, explainable, computationally efficient framework that would be able to effectively identify and explain complex violent and weapon-based behaviors in dynamic urban settings. The main research contributions of this work are summarized as follows:

- We propose DeReV, a novel explainable deep feature-level ensemble framework that integrates two variants from each of the DenseNet, ResNet, and VGG architectures within a multi-backbone feature fusion configuration, enhancing feature diversity, robustness, and classification accuracy for intelligent surveillance systems.
- The framework categorizes the CCTV footage into three separate groups, i.e., Normal, Violence, and Weaponized, based on the SCVD dataset, which allows fine-grained interpretation of the safety situation in cities.
- The framework uses Grad-CAM as a spatial-feature visualization method, LIME as a local interpretability technique for video frame predictions, and counterfactual/temporal importance analysis as a method of identifying the key frames that contribute to the classification results.
- The framework leverages a cloud-edge hybrid environment to enhance predictive accuracy, interpretability, and accountability in AI-driven surveillance to enable citizen-centric safety and proactive threat prevention.

The rest of the paper is organized as follows: Section II provides the related work. In Section III, the proposed framework is described. In Section IV, the performance evaluation is highlighted. Section V presents experiments, results and discussion, and in the last section, Section VI, we conclude the article with several future directions.

## 2 Related work

We conducted a comprehensive review of existing literature focusing on the application of deep learning techniques in smart city surveillance systems. The advent of smart cities has redefined how cities are being administered, tracked, and guarded. The rising population of cities and the rising use of online infrastructures have intensified the demand for smart surveillance systems that have the ability to automatically detect, extract, and react to abnormal activities in real time. Intelligent video surveillance is significant to keeping safety and compliance with regulations as well as to allowing proactive city control. A large-scale bibliometric and technological review was conducted by Sharma and Kanwal (2024) to identify the development of video surveillance technologies in smart cities. Three key goals were identified in the research, namely, emphasizing the relevance and the current trends of intelligent surveillance, providing a standardized model of smart city surveillance, and discussing the drawbacks of existing systems in different spheres. As it was noted in the review, surveillance systems are ubiquitous elements of all subdomains of smart cities, including traffic and public safety and environmental surveillance. The authors found that the rise of new technologies, like deep learning, the Internet of Things (IoT), and computer vision, has made the process of detecting anomalous behaviors, digital tampering, and intruders much easier. The study presented in Myagmar-Ochir and Kim (2023) provided an extensive overview of video monitoring systems, with a particular focus on their in-built application in smart urban infrastructure. The paper brought to the fore the use of deep learning models alongside cloud and edge computing to enable surveillance systems to use extensive visual data at the source and lower the latency and overdependence on bandwidth. It was also explained that the inclusion of blockchain technology is a potentially positive step toward improving the integrity of the data, its access control, and accountability within multi-stakeholder surveillance networks. In spite of these developments, the authors noted that the system heterogeneity, inadequate edge resources, and absence of explainability in AI-based decision-making systems have become a challenge.

To overcome the problem of video transmission efficiency and bandwidth in smart city settings, Zahra et al. (2021) suggested a video encoding model, salient region, to selectively encode the region of interest (pedestrians, vehicles, and road structures) with gross bandwidth conservation and high visual quality. The method of deep learning led to high results such as a reduction in the bit rate and improvement in segmentation accuracy on two test datasets. The system reduced the computational cost by coding only critical parts of the video frames without losing significant surveillance information. This shows that extraction and region prioritization with the help of deep learning can be used to improve efficiency and flexibility in smart city surveillance. The work on the person re-identification (Re-ID) has also been significantly expanded in the research on smart surveillance, as this issue is of paramount significance in the context of the use of the law enforcement system and in safeguarding the population. The concept of an adaptive feature refinement-based deep learning architecture was introduced into (Maqsood et al., 2023) to resolve the issue of person Re-ID on a variety of camera views. The model determined inter-channel relation through spatial attention and channel attention, and a spatial pyramid pooling layer derived multi-scale features of nonuniform input images. The paper has highlighted the significance of the attention mechanism and multi-scale feature extraction in enhancing recognition strength under natural environmental variations.

In addition to recognizing objects or activities, current research has also considered emotion and behavior knowledge as part and parcel of urban surveillance. In Li et al. (2021), the authors used a DeepID convolutional neural network (CNN) to extract emotions and facial expressions and analyze them in a surveillance video feed. Optical flow features and multi-order double cross (MODC) textures were used to capture the minute facial variation with the dynamic light and pose conditions. The framework was able to develop an emotional semantic space on facial expression, showing that emotion-sensitive surveillance can be practiced strengthening the safety of the population, crowd management, and crisis response mechanisms. Affective computing as a part of surveillance brings new levels of situational awareness to intelligent cities. Besides emotional analysis, Khanam and Roopa (2025) discussed the weakness of the conventional anomaly detection methods by introducing a compact hybrid deep learning framework using MobileNetV2 as a tool of spatial feature extraction and Bi-LSTM as a tool of learning the time-sensitivity of the sequence. On the Smart-City CCTV Violence Detection Dataset, the MobileNetV2-BiLSTM model achieved the result of 94.43% in accuracy, whereas the baseline MobileNetV2 model was 90.17% in accuracy. The hybrid model was resistant to variations in illumination and environmental noise with high computational performance and therefore could be used in real-time edge deployments. The findings confirmed the basic assumption that spatial and temporal modeling are critical in detecting the occurrence of violence and anomalies in CCTV videos with accuracy. The literature emphasizes the transformative role of deep learning, edge computing, and hybrid architectures in advancing smart city surveillance systems. Despite enormous improvements, there are still a bunch of open holes, especially in explainability and efficient AI implementation.

## 3 Proposed framework

**Figure 1:**
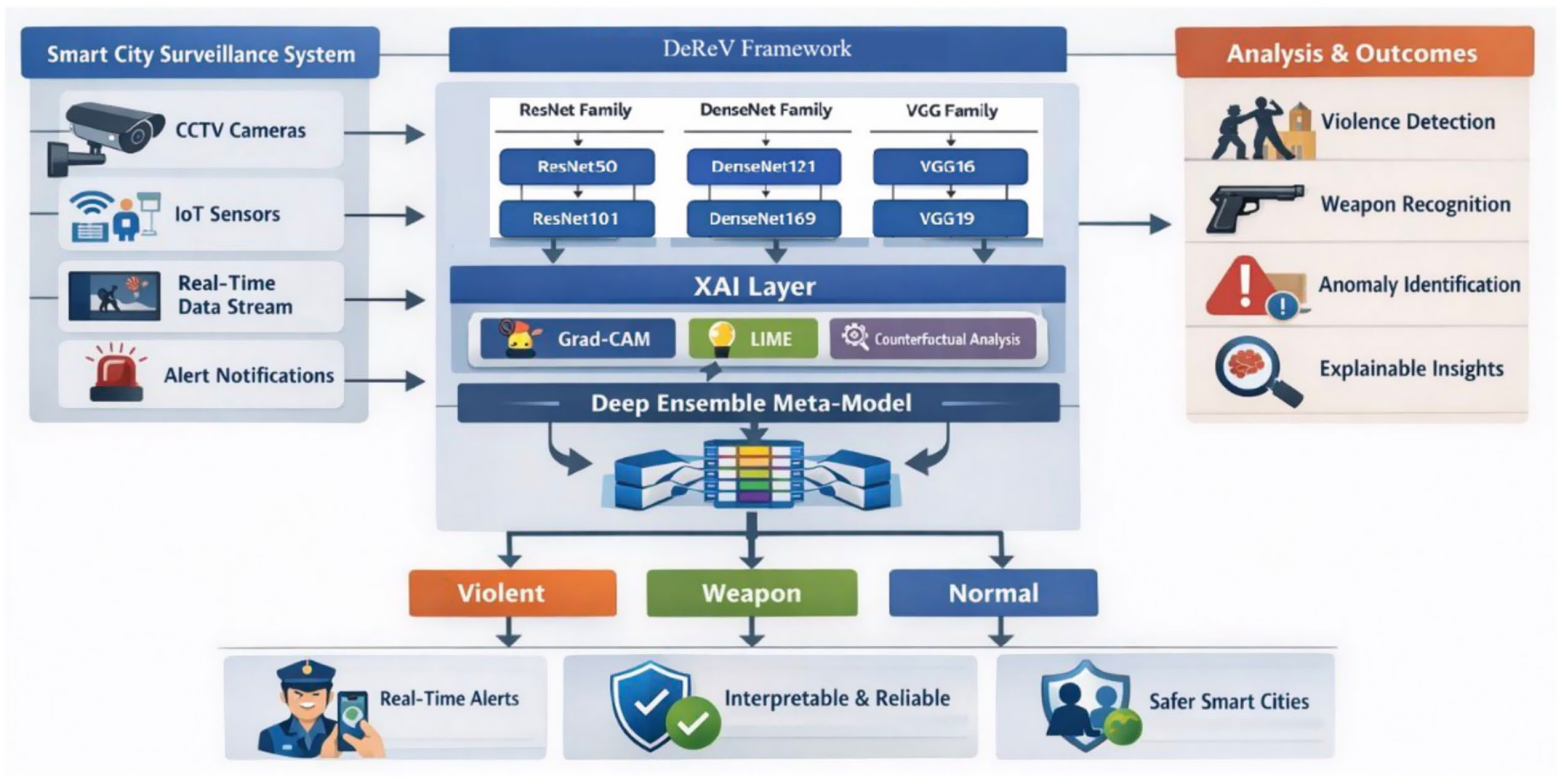
An explainable multi-backbone deep feature fusion framework for intelligent surveillance and citizen safety in smart city ecosystems.

The proposed DeReV framework represents an advanced, explainable deep ensemble architecture designed to enhance intelligent surveillance capabilities in smart city environments. As illustrated in Figure 1, the framework is composed of multiple integrated components that collectively address the challenges of accuracy, interpretability, and transparency in automated violence detection systems.

The DeReV framework presents a novel multi-backbone feature-level ensemble architecture, which integrates the families of DenseNet, ResNet, and VGG architectures. This hybridization discover the distinct advantages of these networks, including feature reuse in DenseNet, residual learning in ResNet, and hierarchical representation in VGG, in order to optimize feature diversity, robustness, and classification accuracy. The feature fusion module of the ensemble concatenates the heterogeneous deep feature embeddings to generate high-confidence predictions. The proposed framework integrates feature embeddings from multiple backbones with varying dimensionalities. ResNet and DenseNet-based streams offer high-dimensional features via temporal pooling, while VGG-based backbones generate compact embeddings using an LSTM layer (128 units). This allows the combination of diverse spatial–temporal information via feature concatenation. The model is used in its working process to classify the CCTV footage under three different categories, i.e., normal, violence, and weaponized. Such discrete sorting provides an opportunity to identify potentially dangerous situations in the city early enough and provides proactive security in the infrastructures of the smart city. The performance of the model is evaluated using Receiver Operating Characteristic (ROC) and Precision-Recall (PR) curves, where a thorough examination of accuracy, sensitivity, and reliability is evaluated under diverse real-world circumstances.

In order to achieve transparency and trust, the framework combines several XAI methods. Grad-CAM is utilized in visualizing spatial attention per frame, which identifies significant areas of attention in model prediction. LIME enables local interpretability, which explains frame-level predictions, whereas counterfactual or temporal importance analysis determines the most influential frames that affect the decision-making process. The processing and inference process is designed to be deployed inside the cloud-edge hybrid space, with the edge nodes performing real-time video analytics and the cloud layer being used to coordinate and aggregate decisions at a higher level. Although the proposed multiple-backbone feature fusion network has a higher computational complexity than single-network approaches, several factors help reduce this overhead. The backbone networks are frozen during inference to skip gradient computations, while the input image size and temporal down-sampling decrease the computational load. These design choices enable the system to be deployed in an edge-assisted environment at near real-time rates. The proposed DeReV framework ensures high predictive capabilities with transparent AI implementation during surveillance scenarios. With interpretability and high detection rates, it will help to achieve citizen-centric safety and preventive response to threats and build secure, intelligent, and trustful smart city ecosystems. The detailed descriptions of each variant are provided below.

### 3.1 RestNet50 and ResNet101

In the proposed DeReV framework, ResNet50 and ResNet101 were built as basic spatial feature extractors to extract complex patterns and neighborhood information on CCTV surveillance video frames. The ResNet family with its residual learning model has proven to address the problem of vanishing gradient in deep neural networks effectively and enables the researcher to extract highly discriminative features despite operating in a complex visual setup. In the paper, both versions were used using pre-trained ImageNet weights to use rich, general-purpose visual representations. The higher-up layers of both networks were removed; only the convolutional backbones were left, and they served as the feature encoders. In order to combine effective training and prevent overfitting, the convolutional layers were frozen, and a Global Average Pooling (GAP) layer was added afterwards to reduce the learned spatial features to condensed high-level features. All the video sequences were run through a TimeDistributed wrapper, which allowed the CNN feature extractor to be run on the frames in the same sequence but with temporal order maintained. The frame-level features were then aggregated, followed by an LSTM layer to include temporal dependencies and motion patterns across frames, which is important in the process of identifying normal, violent, and weaponized activities. Regularization was provided with the help of a dropout layer, and a dense layer that used the softmax activation to perform the multi-class classification was introduced. Convergence stability and training speed were balanced by the optimization of the models with the Adam optimizer with different learning rates.

### 3.2 DenseNet121 and DenseNet169

Following the DeReV framework, DenseNet121 and DenseNet169 were added to the architecture to improve learning spatial representation and address the problem of feature redundancy, which is commonly found in deep convolutional networks. The features of the DenseNet family include the dense connectivity pattern, as every layer has as its inputs all previous layers and features maps to all following layers. This architecture facilitates the use of reuse in features and enhances gradient flow as well as model compactness, which results in efficient learning with limited data. The two versions of DenseNet were set with ImageNet pre-trained weights to make use of generalized visual features and quicken the training process. The highest layers of classification were eliminated in the implemented models, keeping the convolutional feature extraction backbone alone. All the convolutional layers were frozen to achieve overfitting prevention and allow minimization of computational overhead. To reduce the number of spatial features extracted, a GAP layer was attached to compress the features into small global descriptors. These frame-level representations were then passed through a “TimeDistributed” wrapper in sequence so that each frame is extracted by the CNN backbone with the correct temporal sequence. This was then followed by an LSTM layer that was used to learn motion continuity and dynamic temporal dependencies between frames that successfully identified normal, violent, and weaponized actions. The last stages were dropout regularization and a fully connected multi-class SoftMax classifier.

### 3.3 VGG16 and VGG19

The DeReV ensemble also included the VGG16 and VGG19 models, as these rely on deep yet structured hierarchies in the convolutional process to effectively extract spatial features of video frames. Both architectures are known to be simple and highly representational due to the uniform usage of small 3 × 3 convolutional kernels applied sequentially. This design enables them to record finer spatial information and stratified visual features that are important in separating subtle behavioral patterns in camera surveillance, detecting abrupt motions, or identifying weapon-related actions. This paper also uses both VGG models based on ImageNet pre-trained weights to utilize deep-level and mid-level features trained on large-scale natural images. The final fully connected layers were eliminated, and a global average pooling layer was introduced in order to reduce the convolutional outputs to compact feature vectors. As with other base architectures in the DeReV framework, the VGG models were wrapped in a “TimeDistributed” layer to process sequential frames one at a time while maintaining temporal order. The extracted spatial embeddings were then input to a Long Short-Term Memory (LSTM) network, which learned temporal relationships and frame-to-frame motion variations to identify normal, violent, and weaponized scenes. Generalization and multi-class classification were improved by the addition of dropout regularization and a SoftMax output layer, respectively.

### 3.4 DeReV framework

The DeReV framework is the core of this study, and it is aimed at addressing the drawbacks of traditional single-architecture surveillance models by creating a unified multi-backbone feature-level ensemble architecture that integrates multiple deep learning backbones. The name DeReV is derived from the constituent architectures DenseNet, ResNet, and VGG, which are well integrated to take advantage of the complementary capabilities of each in feature representation, generalization, and discrimination. This system makes it possible to achieve the ensemble method since it allows the system to gain a greater understanding of the complex visual and temporal associations that define real-life CCTV images that are frequently affected by changes in illumination, occlusions, and camera viewing angles that are characteristic of an urban environment. The entire six base learners, i.e., ResNet50, ResNet101, DenseNet121, DenseNet169, VGG16, and VGG19, were first trained on the large-scale ImageNet dataset and then immediately adapted to the Smart-City CCTV Violence Detection (SCVD) dataset. These are good generic models of features, and each of them is directed at visual abstractions of different types. The ResNet family receives residual learning characteristics in the way that the gradient propagation is consistent and training functions effectively in a number of layers, which is essential to reveal the faintest alterations of movement in violent events. The DenseNet architecture also reuses features more and allows more gradient flow through dense connectivity, which lets the network learn finer interactions between features and smoother transitions between frames, unlike ResNet. VGG versions create a tradeoff between simple architecture and powerful spatial feature extraction with uniform 3×3 convolutional kernels that are convenient in small-scale object textures and edges to differentiate between normal, violent, and weapon-oriented behavior. The penultimate layer of rich, high-level spatiotemporal embeddings is extracted after each of the base models has processed the video sequences. These embeddings are then added to build a general feature space that pools the information of all the six networks. Such a combination guarantees the local and global visual features are captured, and therefore, the model is more resilient in a range of surveillance scenarios. The concatenated features are followed by a dropout layer (dropout = 0.5) to minimize overfitting and maximize model generalization, followed by a dense layer with SoftMax activation to give the final classification of three categories: normal, violence, and weaponized.

The fusion classifier was trained using the Adam optimizer with the conservative learning rate equal to 1e-5 so that the ensemble model could converge slowly and steadily. In training, each frozen base model brings in fixed deep representations, which gives the fusion layer the freedom to identify the best combinations of features and decision boundaries with the help of multi-architecture knowledge. This strategy is more predictive in addition to the fact that it enables the model to be both computationally efficient and scalable to real-time smart city surveillance systems. The performance analysis confirmed the quality of the DeReV framework, where there was a total accuracy of 96.15 with a balanced performance in all the classes. Specifically, the system scored 100 percent accuracy and 98.08 percent recall on normal activity and 97.96 percent and 92.31 percent accuracy and recall on violent and weaponized activity, respectively. These results make it clear that the framework is able to recognize not only obviously violent or armed behavior but also covert motion clues that can be used to differentiate between aggressive behavior and a normal human motion. In essence, DeReV offers an accurate, readable, and measurable solution to smart city ecosystems in terms of smart surveillance. Its ensemble structure is sturdy and impervious to varied environmental conditions and behavioral environments, and its explainable AI extensions (Grad-CAM, LIME, and Temporal Importance) ensure transparency in decision-making.

### 3.5 Algorithm for the proposed framework

The proposed framework is presented in Algorithm 1, which shapes the whole working process of the DeReV framework. It takes a combination of deep learning and explainable AI to act as intelligent surveillance and identify various types of violence. It begins by preprocessing video and frame sampling, where raw CCTV footage is run through to generate representative frames. All the images are standardized and scaled to be of equal size to reduce noise levels in them and to be present in a normal form to be introduced into deep models. In the feature extraction step, the framework takes three various CNN backbones, that is, ResNet, DenseNet, and VGG, to obtain complementary spatial features of video frames. All these architectures add hierarchical feature representations, thereby adding diversity and strength to learned features. Such frame-level embeddings are then input to a temporal encoding module based on the Long Short-Term Memory (LSTM) networks to receive training on temporal relationships between frames and comprehend temporal dynamics of violent behaviors.

The three CNN-LSTM streams are then coded and concatenated and form a fused feature that is a global collection of multi-tiered spatiotemporal data. This composite representation is then fed into a fusion classification layer, which uses a fully connected network and is further topped with a SoftMax activation to approximate the probability of the three categories (normal, violence, and weaponized). The model parameters are optimized with the help of categorical cross-entropy loss that guarantees the correct multi-class discrimination. In order to interpret the AI and make it transparent, the XAI techniques are also integrated into the framework. Class-discriminative heatmaps are obtained using Grad-CAM and indicate spatial regions in the frames that are most predictive. LIME offers local interpretability by analyzing the effect of perturbations in frame segments on the result of the classification, whereas temporal counterfactual analysis determines important frames that most significantly influence the decision made by the model. The XAI modules provide a descriptive insight into model reasoning, which makes human beings understand and trust AI-based surveillance systems.

ALGORITHM 1 DeReV framework: an explainable multi-backbone deep feature fusion framework.
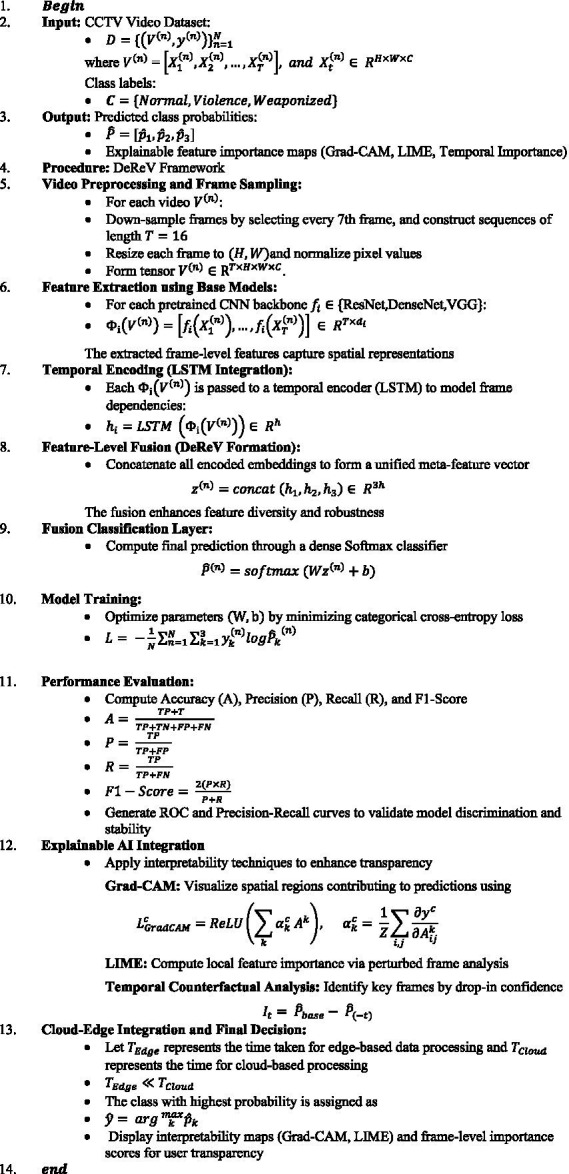

## 4 Performance evaluation

To comprehensively assess the effectiveness of the proposed framework, the performance evaluation process is structured into three key components. The first component is the evaluation parameters, which define the metrics used to measure model accuracy and reliability. The second component is the dataset, which provides the foundation for training and validation. The third component is the dataset preprocessing, which ensures data consistency and quality for optimal model performance.

### 4.1 Performance evaluation parameters

To ensure that the proposed DeReV framework was effective, a series of quantitative performance measures and graphical analyses were used to properly evaluate the effectiveness of the measures and ensure the findings were precise, valid, and interpretable. It was analyzed in terms of accuracy, precision, recall (sensitivity), F1-score, confusion matrix, ROC curve, and PR curve. Accuracy The overall percentage of true video instances in all the classes. It provided a pre-test of the ability of the proposed model in dealing with various surveillance scenarios. Precision and recall were also used to evaluate the models to determine the extent to which they are trustworthy in the selection of critical events. Precision calculates the proportion of true positive alarms of all true positive predictions and demonstrates the frequency of alarms of the violent or weaponized behavior of the model. As mentioned, our sensitivity is a metric that quantifies the ability of the model to identify all the (relevant) instances of each category, and violent or weaponized behavior is not excluded when doing real-time observation. A summation of these measures gives the foundation of the F1-score, which is the harmonic mean of precision and recall. The F1-score is a single, in-between measure of the classification accuracy that is particularly useful where the cost of a false negative (missed violent incidents) is high. Moreover, ROC and PR curves were also used to assess the discrimination power of models and threshold behavior in addition to these numerical metrics. The ROC curve is a graph that shows the true positive (recall) rate versus the false positive rate of all decision thresholds, and the area under the ROC curve (AUC) provides the general classification accuracy. Large AUC is a sign of better separability across classes. Imbalanced datasets are better presented in a PR curve, which gives the trade-off between the precision and the recall at given thresholds.

### 4.2 Dataset

The dataset used in the present research work is publicly available on Kaggle platform with the title, “Smart-City CCTV Violence Detection Dataset (SCVD)” (Kaggle, 2025). It is a filtered set of surveillance video clips that were chosen with the intent of use in studies of violence detection and weaponized incident detection in urban CCTV scenes. The dataset reflects three different behavioral categories, one of which is Non-Violent (non-violent activities in a daily environment), Violence (physical confrontations, assaults, violence), and Weaponized (incidents that involved the use of a weapon or other weapon-like objects). SCVD offers trimmed clips of CCTV-like vantage points and thus is more realistic of urban surveillance conditions than most other datasets of violence detection, which tend to use sport videos or those of handheld cameras. It is structured in training and testing subsets in their own folders to allow researchers to adhere to a similar experimental procedure. As an illustration, there are about 2,746 training videos and 477 testing videos in all three classes, as reported by a single source. Regarding data preparation, each video clip is already in a manageable format; this is why preprocessing of deep-learning models is made easier. The introduction of a separate Weaponized category offers an in-between category to the existing datasets, as many have historically not captured direct instances of weapon carrying or weapon-related behaviors. The SCVD was designed to enable the identification of both weaponized and non-weaponized violent instances to be useful in the application of smart cities, which require a fine-tuning of the recognition of threats. The SCVD dataset offers a practical, domain-relevant benchmark for intelligent surveillance research in smart urban environments.

### 4.3 Dataset preprocessing

The dataset has three different classes of surveillance video clips: normal, violence, and weaponized activities. The preprocessing pipeline was tailored so that it would provide consistency, computational efficiency, and adequate data diversity in order to increase the learning and generalization abilities of the model. To make the working environment, the necessary libraries were imported: TensorFlow, OpenCV, NumPy, ImgAug, and scikit-learn. In order to replicate the results of several runs, the TensorFlow random seed was set to a fixed value. Although TPU initialization was to be supported to facilitate high-performance training, the default in this paper was to use the GPUs. A clean and orderly data pipeline was achieved with the help of the directory management functions, and the version and the use of GPUs in TensorFlow were checked and their status verified in advance before launching the preprocessing sequence.

The dataset was initially separated at the original video level using a stratified split (80–20) to guarantee rigorous assessment and avoid data leakage after which temporal division was made. This ensures that every frame sequence of a particular video will only be found either in the training set or testing set, but not both. Following this video-level division, frame sampling, data enriching, normalization, and time bundling were performed individually in each subset. This rigid division negates all the chances of temporal or scene-level information leakage and guarantees the validity of the reported performance measures. The “videotoframes()” function has been created with the help of OpenCV to read videos and sample frames and ignore unnecessary ones in order to minimize duplication and computation costs. Frames are first down sampled by selecting every seventh frame from the original video to reduce redundancy. From these sampled frames, sequences of 16 frames are constructed to represent temporally distributed segments.

**Figure 2:**
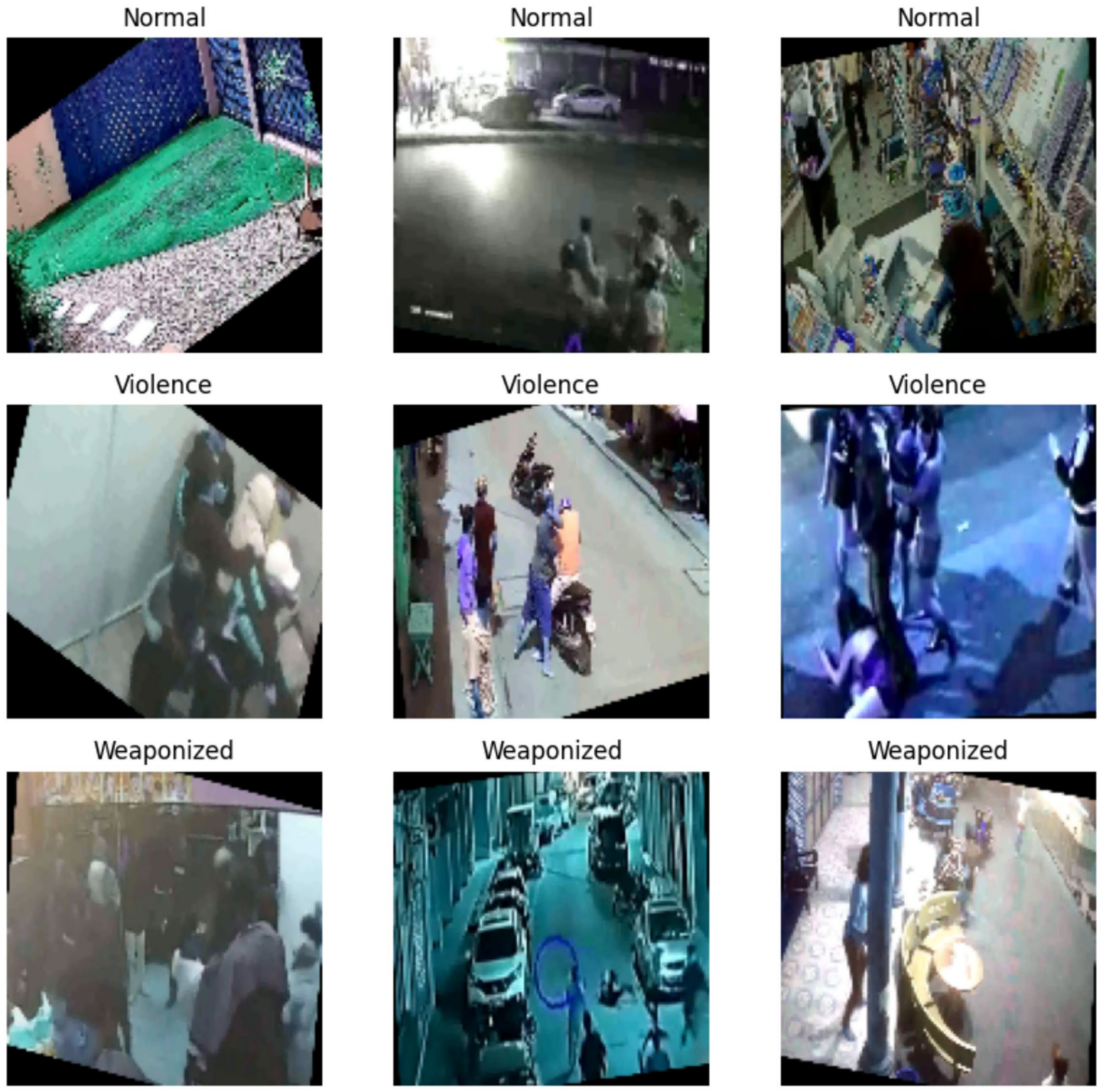
Sample frames from each class illustrating the visual diversity of the SCVD dataset.

At this step, data augmentation methods were used in succession exclusively on the training subset to augment data and minimize overfitting with the help of the “imgaug” library. They included horizontal flipping, zoom, and random change of brightness and rotation within the range of ±25°, so that the model can adapt itself to light variations, changes in perspectives, and object position in a variety of surveillance systems. All the augmented frames were converted to RGB and down sampled to 128 × 128 pixels to ensure that the input size used was consistent across the ensemble models (DenseNet, ResNet, and VGG). After extraction and augmentation of the frames were done, the dataset was extracted into arrays of images (X-original) and their class labels (Y-original). Sampling was only used in the training set to equalize representation of video clips per category so that there was equal representation of normal, violent, and weaponized categories. As Figure 2 presents, several sample frames of every class demonstrate the visual diversity of the dataset of SCVD, illustrating the variety of the crowd scenes, objects present in them, and movement patterns between the three types of activities.

The raw video frames were then converted into NumPy arrays in order to facilitate easy processing. The data was adjusted to be in a format of input to the deep learning model by performing pixel value division by 255 to bring them into the range of [0, 1]. Each frame was reshaped into a four-dimensional array, where one dimension was set to (128, 128, 3) to represent the image height, width, and RGB color channels. Since the dataset included videos and not individual images, there was a temporal structuring step that was conducted to organize the successive frames into brief video segments. A constant number of 16 frames per video segment was chosen to capture spatial and temporal information. The final tensor structure was (N, 16, 128, 128, 3), where *N* represents the total number of video sequences. Each 16-frame sequence directly inherited the original label of its source video to maintain temporal consistency of labeling. One-hot encoding of the target labels was performed with the TensorFlow “to_categorical()” method in order to transform the class labels into categorical matrices to be used in multi-class classification. The resulting datasets consisted of training and testing videos derived strictly from the initial video-level split, each labeled in one of the three classes (normal, violence, or weaponized).

## 5 Experiments, results and discussions

This section presents the experiments, results, and discussions conducted to validate the effectiveness of the proposed framework. The experimental analysis is structured to provide a comprehensive evaluation across multiple dimensions. The results of ResNet, DenseNet, and VGG variants are discussed individually to establish comparative baselines, followed by the performance analysis of the proposed DeReV framework. Interpretability analyses using Grad-CAM, LIME, and temporal counterfactual explanations are presented to provide insights into model decision-making.

### 5.1 Performance comparison

A comparative analysis of the classification performance for individual deep learning models, ResNet50, ResNet101, DenseNet121, DenseNet169, VGG16, and VGG19, against the proposed DeReV framework is presented in Table 1. The measures of evaluation are Precision, Recall, F1-score and Accuracy on three activities categories: Normal, Violence, and Weaponized. This overall analysis shows that DeReV model has a better result than the other underlying models in obtaining higher accuracy, consistency, and balance of all performance measures. Of the single architectures, ResNet50 and ResNet101 were found to have strong feature learning properties with the overall accuracy of 90.38 and 91.66, respectively. ResNet50 was able to be perfectly accurate on the Normal category (1.0000) although it had lower values on Violence and Weaponized classes with precision scores of 0.8571 and 0.8679, respectively. ResNet101 had a better accuracy on the Weaponized category at 0.9767, indicating its increased capability at capturing complex items and motions. Nonetheless, both versions did not have a consistent performance across all classes, which suggests that there was some bias to visual patterns, which may influence their validity in a real-life smart surveillance environment.

**Table 1:** Comparative performance analysis.

Models	Metrics	Class			Macro average	Weighted average
		Normal	Violence	Weaponized		
RestNet50	Precision	1.0000	0.8571	0.8679	0.9084	0.9084
	Recall	0.9038	0.9231	0.8846	0.9038	0.9038
	F1-score	0.9495	0.8889	0.8762	0.9049	0.9049
	Accuracy	0.9038461538461539				
ResNet101	Precision	0.9434	0.8500	0.9767	0.9234	0.9234
	Recall	0.9615	0.9808	0.8077	0.9167	0.9167
	F1-score	0.9524	0.9107	0.8842	0.9158	0.9158
	Accuracy	0.9166666666666666				
DenseNet121	Precision	0.8868	0.7903	0.9756	0.8842	0.8842
	Recall	0.9038	0.9423	0.7692	0.8718	0.8718
	F1-score	0.8952	0.8596	0.8602	0.8717	0.8717
	Accuracy	0.8717948717948718				
DenseNet169	Precision	0.9273	0.8814	0.9762	0.9283	0.9283
	Recall	0.9808	1.0000	0.7885	0.9231	0.9231
	F1-score	0.9533	0.9369	0.8723	0.9208	0.9208
	Accuracy	0.9230769230769231				
VGG16	Precision	1.0000	0.8727	0.9245	0.9324	0.9324
	Recall	0.9231	0.9231	0.9423	0.9295	0.9295
	F1-score	0.9600	0.8972	0.9333	0.9302	0.9302
	Accuracy	0.9294871794871795				
VGG19	Precision	0.9388	0.7538	0.9524	0.8817	0.8817
	Recall	0.8846	0.9423	0.7692	0.8654	0.8654
	F1-score	0.9109	0.8376	0.8511	0.8665	0.8665
	Accuracy	0.8653846153846154				
DeReV	Precision	1.0000	0.9796	0.9107	0.9634	0.9634
	Recall	0.9808	0.9231	0.9808	0.9615	0.9615
	F1-score	0.9903	0.9505	0.9444	0.9617	0.9617
	Accuracy	0.9615384615384616				

The variants of DenseNet showed the high feature reuse and gradient flow benefits and gave more balanced results. DenseNet121 was the best with an accuracy of 87.18, and high precision in Weaponized events (0.9756), but the recall is relatively lower (0.7692), which means that the model fails to produce results in the cases of weapon-like objects being obscured or under-illuminated. DenseNet169 in its turn provided a more stable result with the accuracy of 92.30, the best among the individual DenseNet models. It showed complete memorization of Violence (1.0000) and high accuracy of the Weaponized actions (0.9762) thereby confirming its high ability to generalize over time limits of actions and detect objects in crowded scenes. Competitively performed was the VGG family whose architectural simplicity and high extraction of spatial features make it a competitive choice. VGG16 had the best result with an accuracy of 92.95, which was better than all the individual models except DenseNet169. It gave the ideal accuracy in the Normal class and in all categories, the precision and recall was well-balanced, indicating its capability in texture and contour-based feature extraction. Although VGG19 was slightly less accurate (86.53%), it was a reliable performance with a decrease in Violence (0.7538) and Weaponized (0.9524) classes, which is why further VGG architecture might add redundancy with no strong enhancements on generalization in this dataset.

The proposed DeReV architecture outperformed all individual baselines, achieving an overall accuracy of 96.15% and macro/weighted average F1-scores of 0.9617. The class-wise precision values were 1.0000 (normal), 0.9796 (violence), and 0.9107 (weaponized), while recall values were 0.9808, 0.9231, and 0.9808, respectively. The high recall for Violence and Weaponized categories indicates strong sensitivity and reduced false negatives, which is critical in safety-sensitive surveillance applications where missed detections can have severe consequences. The strategy of multi-backbone feature fusion can be credited with performance improvement. The framework builds on the temporally encoded embeddings of more than one pretrained network to construct a richer and more varied feature space than any one model. The SoftMax classification layer is the final layer, which acts based on this single representation, and it learns discriminative decision boundaries in the fused feature space. In contrast to decision-level ensembling, this methodology does the integration at the representation level, with the ability to optimize both spatial and temporal cues across architectures. The proposed framework achieves the highest total accuracy and F1-score, and, as shown in Table 1, it is balanced in all activity classes. This uniformity is a validation of its strength and applicability to real-time smart surveillance systems that can support a scalable and intelligent safety platform within the smart city environment.

**Figure 3:**
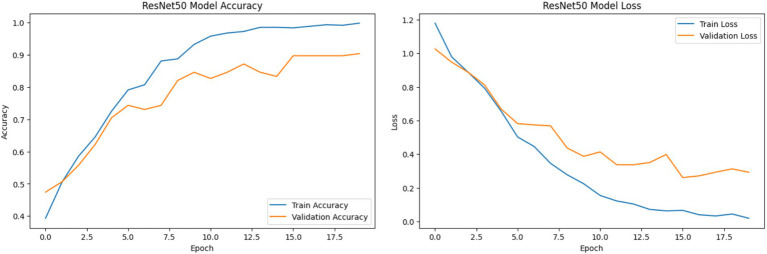
Model accuracy and loss for ResNet50.

**Figure 4:**
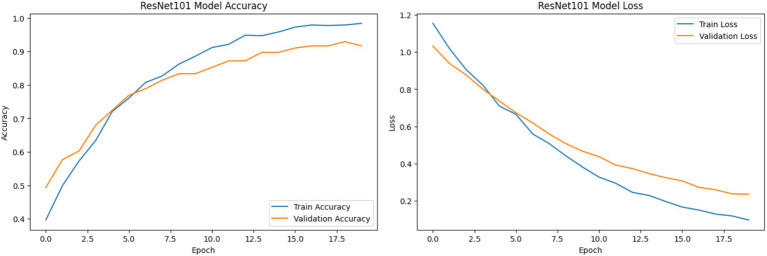
Model accuracy and loss for ResNet101.

**Figure 5:**
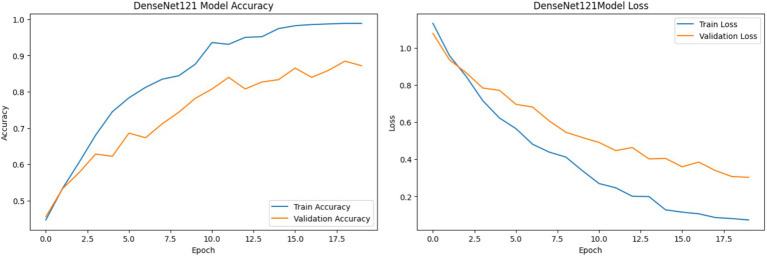
Model accuracy and loss for DenseNet121.

**Figure 6:**
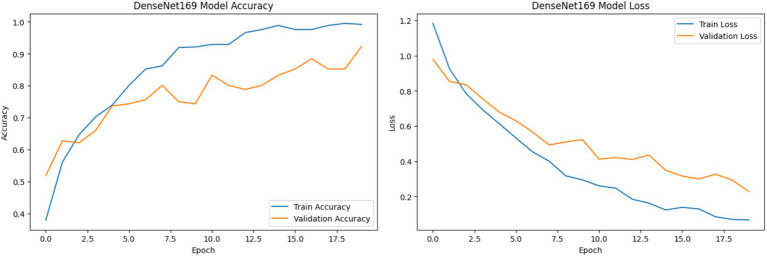
Model accuracy and loss for DenseNet169.

**Figure 7:**
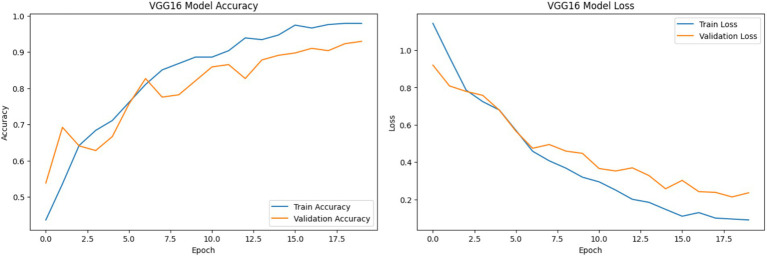
Model accuracy and loss for VGG16.

**Figure 8:**
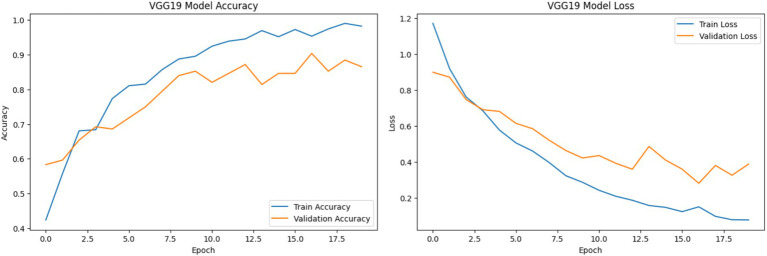
Model accuracy and loss for VGG19.

**Figure 9:**
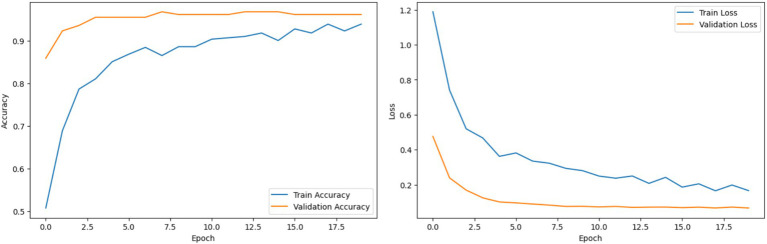
Model accuracy and loss for DeReV.

Performance in training and validation of the individual CNN models are compared against the proposed DeReV framework in Figures 3–9. Each figure presents two plots: the model accuracy curve (left) and the model loss curve (right), illustrating performance progression across training epochs. Figure 3 indicates that ResNet50 has a gradual accuracy gain that levels off to a steady value of 0.90 as the loss steadily decreases, which suggests that it has stable convergence behavior. Figure 4 (ResNet101) has a little better generalization, as it has better validation accuracy and lower validation loss than ResNet50. This is probably because the deeper architecture allows representing hierarchical features better.

The results of DenseNet121 and DenseNet169 are provided in Figures 5, 6, respectively. Both models have a smoother convergence curve and a lower difference between training and validation performance. Specifically, DenseNet169 is faster to converge and has less overfitting, which can be explained by the superior gradient propagation as well as feature reuse mechanisms due to the dense connectivity. Figures 7, 8 show the performance of VGG16 and VGG19. VGG16 is able to maintain a constant level of accuracy and has a low discrepancy between training and validation loss, pointing to good generalization. In comparison, VGG19 reveals that there is slightly more divergence in the loss curve, which signifies that there is slight overfitting. However, the two architectures are competitive, which proves the usefulness of VGG-based models in visual classification using discriminative spatial features. Lastly, Figure 9 shows the training dynamics of the proposed DeReV framework. The overall validation accuracy of the model is the best, and its loss convergence is found to be the most stable compared to other considered architectures. The similarity between the training and validation curves denotes better generalization and less overfitting. Although feature concatenation increases dimensionality, the use of pretrained frozen backbones as well as temporal feature abstraction and dropout regularization effectively controls overfitting. The consistency between training and validation performance further supports the generalization capability.

The increased stability and the decreased fluctuations of the loss in the proposed framework can be explained by the multi-backbone feature fusion approach. The temporally encoded representations of the ResNet, DenseNet, and VGG structures are fused into a single feature space, and then final classification is made. This level of integration is at the representation level, where complementary spatial and temporal characteristics can be optimized together in a single SoftMax classifier. The convergence trend and the reduced loss of validation prove that the DeReV framework has better generalization and learning stability, which validates the presence of a suitable model in addressing various and complex surveillance video conditions.

**Figure 10:**
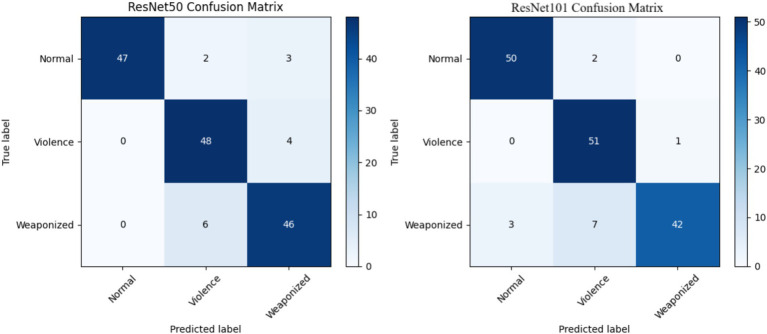
Confusion matrices for ResNet50 and ResNet101.

**Figure 11:**
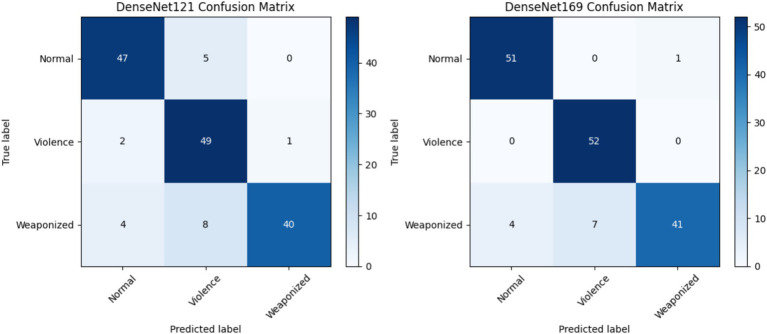
Confusion matrices for DenseNet121 and DenseNet169.

**Figure 12:**
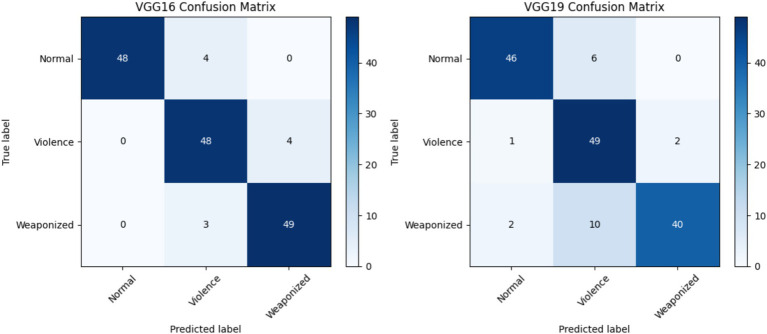
Confusion matrices for VGG16 and VGG19.

**Figure 13:**
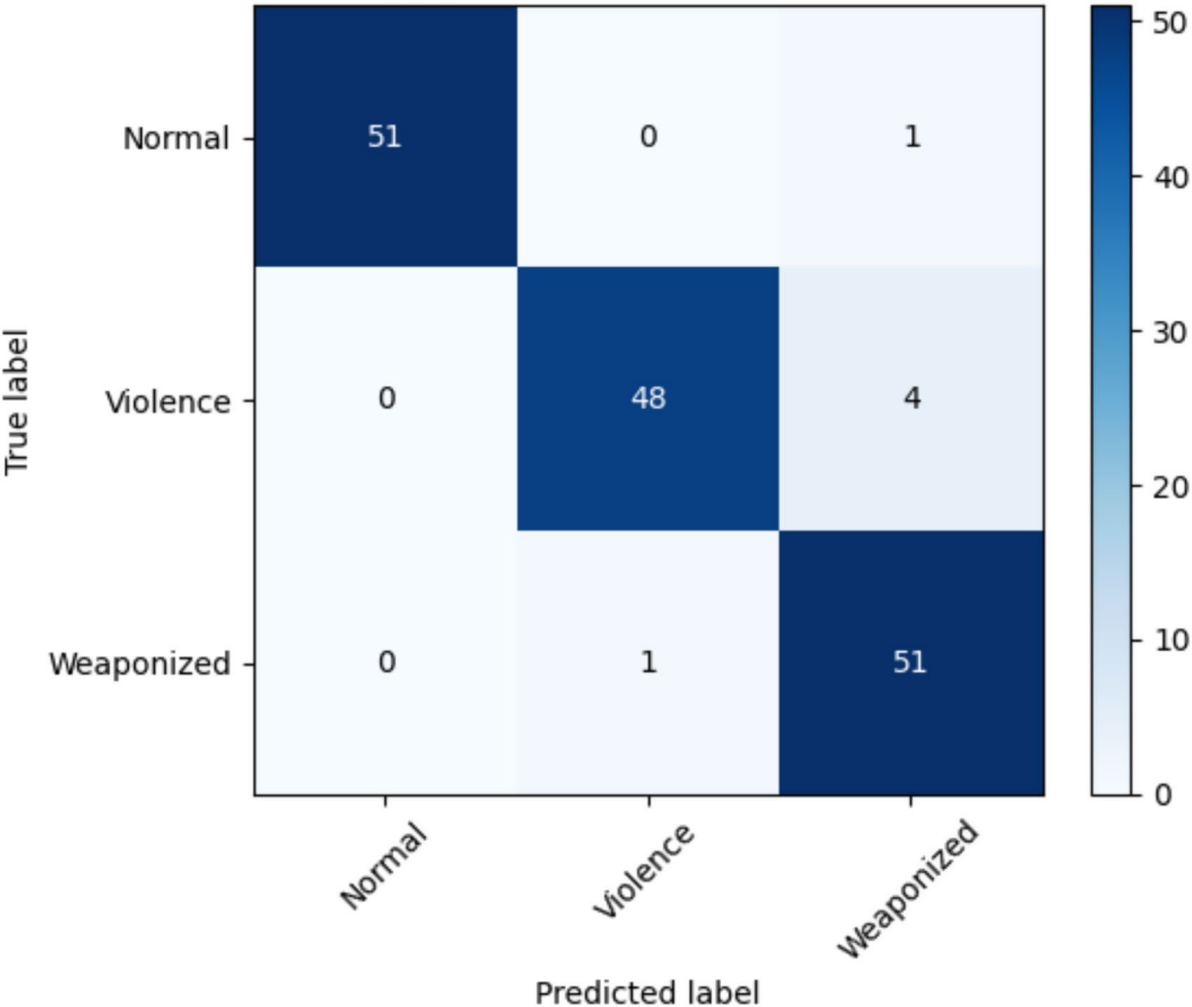
Confusion matrix for DeReV.

A more detailed analysis of how the different deep learning models evaluated performed in the three-class problem: normal, violence, and weaponized is presented in the confusion matrices of the evaluated models in Figures 10–13. Figure 10 (ResNet models) depicts that ResNet50 and ResNet101 perform well with the two categories that are Normal and Violence but find it challenging to identify the Weaponized category. Specifically, both models classify a number of weaponized samples incorrectly as normal (3 cases each), and ResNet101 also classifies 7 weaponized samples as violence. This implies that residual-based architectures, though useful at capturing motion and confrontation patterns, might not be completely reliable to distinguish unobtrusive object-level cues that can distinguish between weaponized and general violent behavior.

The DenseNet models (DenseNet121 and DenseNet169) exhibit a more balanced classification behavior as reflected in Figure 11. DenseNet121 shows quite stable results with few cases of misclassification between the three classes. DenseNet169 gets good results in the Normal category and Violence category but is also confused with Weaponized cases and wrongly classifies 7 Weaponized cases as Violence. This indicates that, as the connectivity density increases, propagation of features and depth of representation increase; however, subtleties in inter-class differences between violence and weaponized behavior are difficult to capture in individual backbone models. Figure 12 shows competitive and stable results of the confusion matrices of VGG16 and VGG19. VGG19 generates the most stable classification scores among all three classes (49 samples of each category were correctly classified). The misclassification rates of both types of VGG are relatively low and uniformly distributed in contrast with ResNet and DenseNet models. Nevertheless, there is still a bit of confusion between the Violence and Weaponized categories (e.g., VGG19 confuses 1 Violence instance of Weaponized and 1 Weaponized instance of Violence), indicating the fact that these two types of activities are similar to each other. Lastly, Figure 13 shows the confusion matrix of the proposed DeReV framework, which performs best in both classification accuracy and balance in the class-wise performance as compared with all the methods considered. The framework minimizes the misclassification errors greatly with a perfect classification of the “weaponized” category. There are only two instances of misclassification in general: one instance of a normal that should have been classified as violence and one instance of violence that should have been classified as weaponized. The improved results of the framework are because of the multi-backbone feature-level fusion approach that combines complementary spatial representations of ResNet, DenseNet, and VGG architectures. The framework improves the separability of classes by building a unified and enriched feature space before the eventual classification, which improves separation between the two similar categories, Violence and Weaponized. The confusion matrix analysis supports the fact that the effective approach achieved better robustness, less inter-class confusion, and higher reliability when identifying a multi-class surveillance activity, thereby being highly suitable in the most complex real-world situation of smart city monitoring.

To evaluate the appropriateness of the proposed DeReV feature-fusion architecture to real-time video inference in smart surveillance settings, the trained model was experimented on unseen video sequences. To be consistent and computationally efficient, each video was uniformly sampled to obtain 16 frames in his or her temporal span. This approach allows the model to streamline critical movement and activity patterns and minimize redundancy and the inference cost. The extracted frames were resized to 128×128 pixels and normalized with a preprocessing function that would work with all the convolutional backbones (ResNet, DenseNet, and VGG variants). It is a standardized preprocessing that guarantees consistency in model performance across lighting conditions, backgrounds, and crowd dynamics that may occur in urban surveillance scenarios.

The preprocessed sampled frame sequences were then processed through the DeReV architecture, which combines the spatial representations of six pretrained CNN backbones (ResNet50, ResNet101, DenseNet121, DenseNet169, VGG16, and VGG19) and then temporally encoded with LSTM layers. All the backbones perform the extraction of complementary spatial features, with the temporal encoder recording the motion dynamics and frame-to-frame dependencies. All the backbones feature embeddings that are temporally encoded and concatenated to create a single representation, which is then fed through a final SoftMax classification layer. The model provides probability scores of the three defined classes, namely, Normal, Violence, and Weaponized. The most probable class is picked as the final prediction, and the associated confidence score is taken.

**Figure 14:**
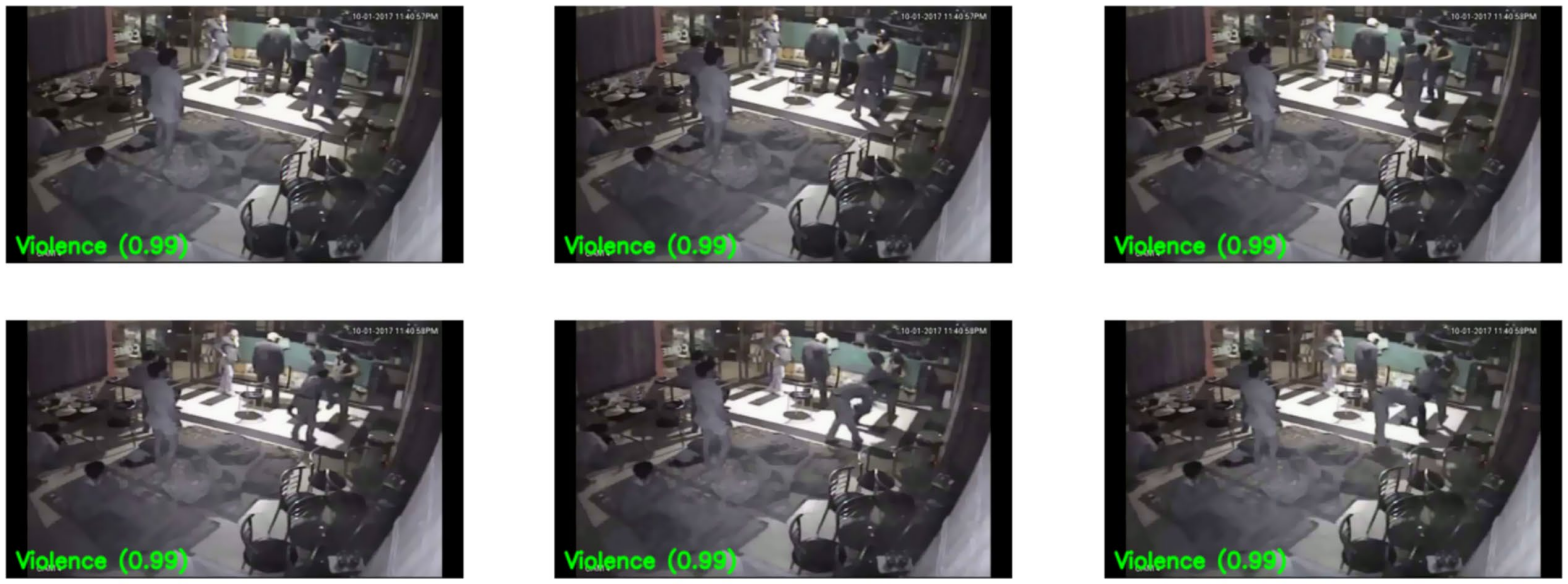
Representative frames sampled from a video clip.

To visualize the given outcomes, the predicted class label and confidence score are superimposed on the frames of the video, and one can intuitively interpret the decisions made by the model. Figure 14 shows one of the inference results with six representative frames of a test video. In case in point, in the model, the sequence is recognized as violence with a confidence score of 0.99, and the chances of being normal and weaponized are insignificant. The aggressive motion pattern of the model is evident through the selected frames, and one can observe that it recurs through time. The overlay of the green text with a prediction of the class and a level of confidence increases the interpretability and helps it be deployed in real-time settings. This qualitative analysis shows that the offered DeReV framework can effectively apprehend the temporal coherence and identify subtle dissimilarities between the normal, violent, and weaponized ones, which proves its appropriateness to actual smart surveillance systems.

**Figure 15:**
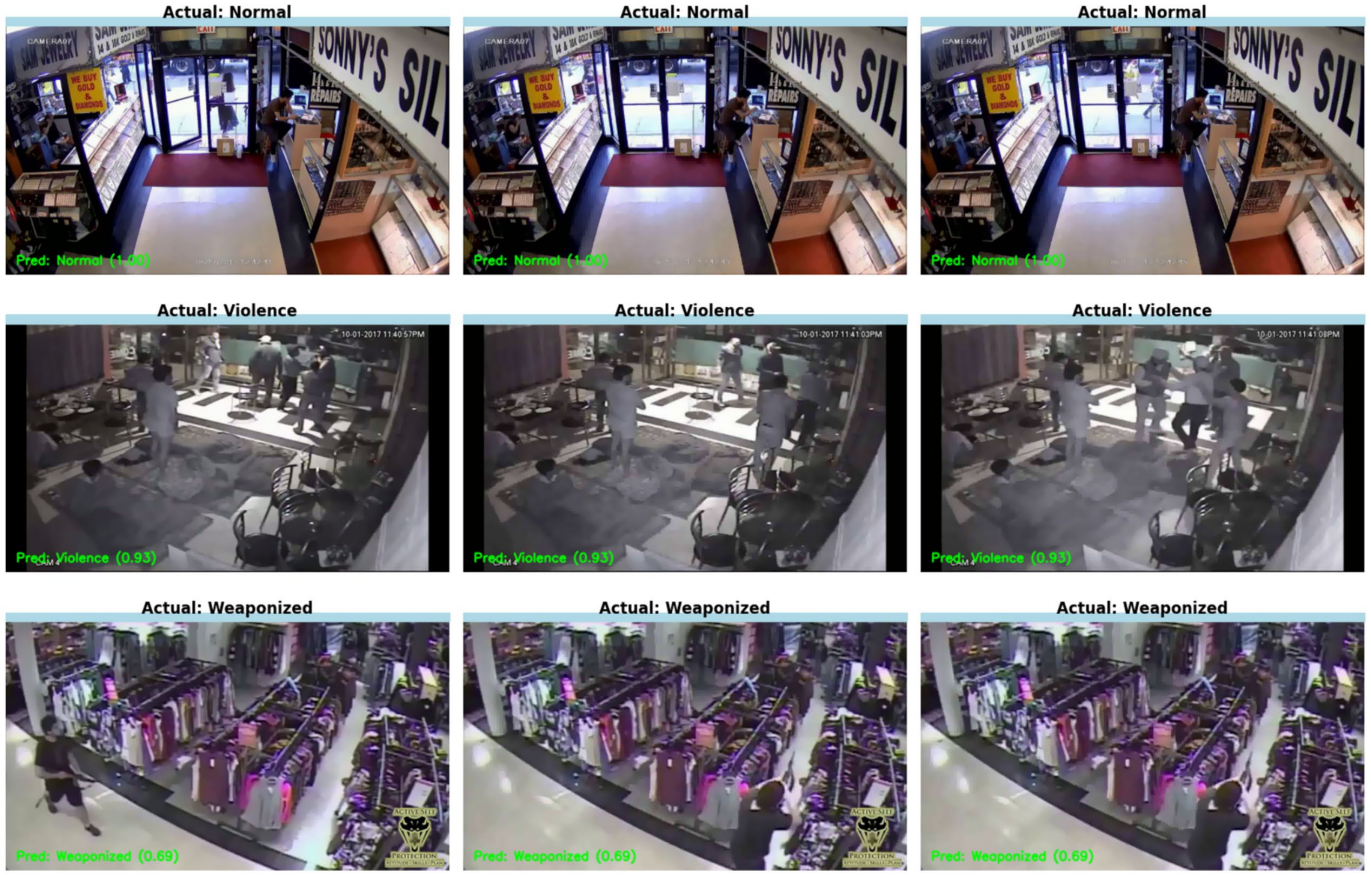
Multiclass visualization using representative test videos from the SCVD dataset.

A visualization experiment on appropriate samples of test videos based on various types of activities was also performed to show the capability of the proposed DeReV framework to classify and generalize the data. The aim was to measure the interpretability of the model as well as the capacity of the model to differentiate the behavioral patterns of normal, violent, and weaponized in the real-life scenario of urban surveillance. In this experiment, each of the classes had one representative video that was processed with the trained DeReV model. Every video was subjected to the standardized temporal sampling process whereby 16 frames were sampled out in a uniform manner throughout the duration of every clip. This was done to provide sufficient representation of both motion dynamics and stationary contextual features. The individual extracted frame was rescaled to an image size of 128 × 128, then normalized through ImageNet-based preprocessing, and then submitted to the model to run inference. The probability distribution of the three classes was obtained, and the predicted label of the classes was chosen using the highest probability value and its associated confidence value. In order to achieve a visual summary that can be interpreted, a visual summary of three representative frames per class was obtained by selecting and labeling them by the predicted labels and confidence values over the top of the images. This multi-class visualization experiment has been found to produce the following results, as shown in Figure 15. The first row is associated with normal activity, where the model is able to identify non-aggressive human behavior and stable background dynamics with high confidence. The second row is violence scenarios, which can be described as violent interactions and physical confrontation. When used in such situations, the model shows high confidence in violent behavior detection using motion intensity as well as spatial interaction as indicators. The third row demonstrates weaponized actions, such as the presence of an object, such as a knife or a gun.

**Figure 16:**
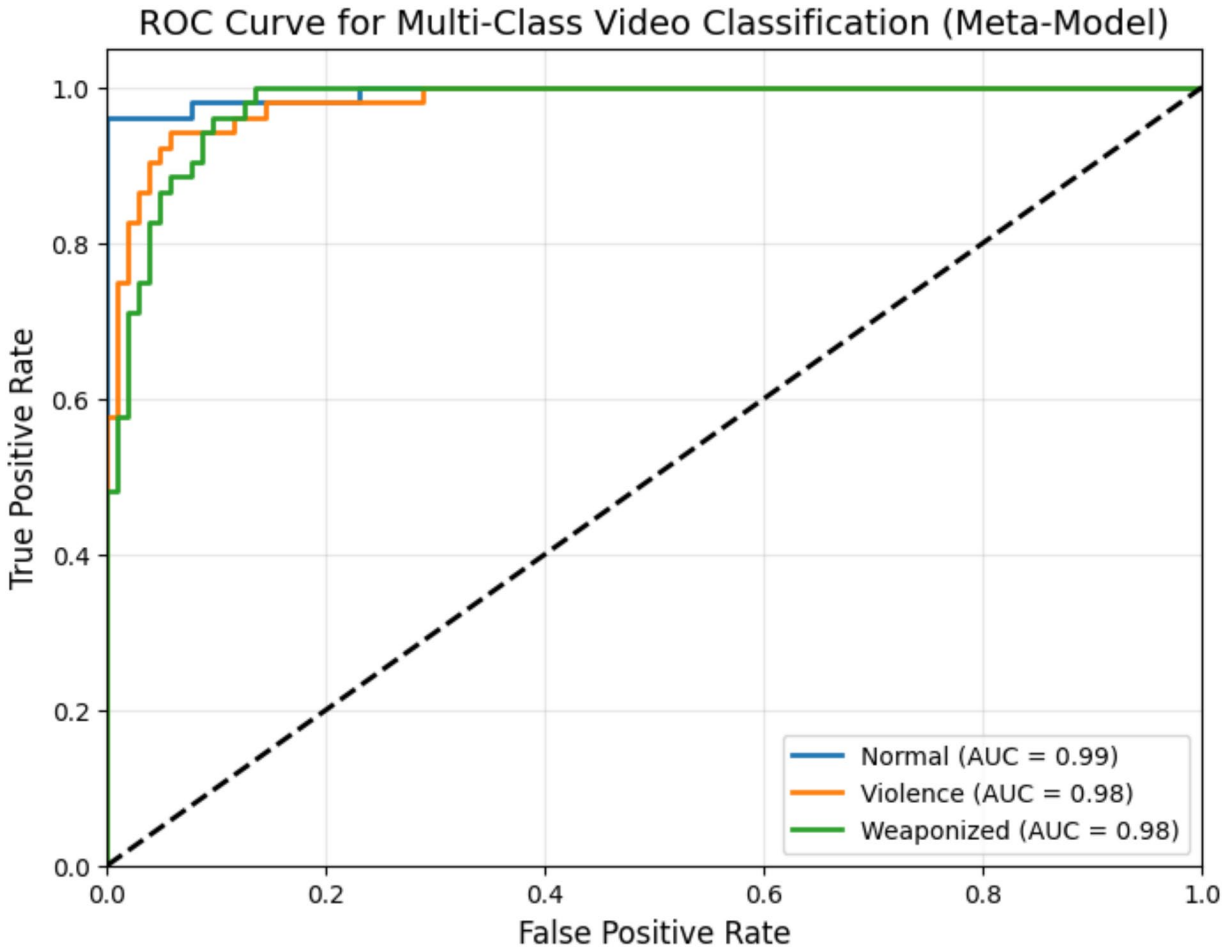
ROC curve for DeReV.

In order to further scrutinize the discriminative performance of the proposed DeReV architecture using more than the traditional measures of accuracy and F1, a Receiver Operating Characteristic (ROC) analysis was conducted on all three behavioral ones, i.e., Normal, Violence, and Weaponized. Thanks to the use of the ROC curve, the model, in contrast to a basic classification model, is able to effectively differentiate between the positive and negative examples of each class by using the True Positive Rate (TPR) in relation to the False Positive Rate (FPR) at different classification thresholds. This ability to discriminate is quantified by the values of the corresponding area under the curve (AUC), the larger the AUC, the greater the ability of the classifier. As Figure 16 shows, the DeReV ensemble model has a superior classification reliability level. Precisely, the model had an AUC of 0.99, 0.98, and weaponized activity classes had AUCs of 0.98 and 0.98, respectively. The almost-ideal ROC profiles mean that the suggested architecture is effective at reflecting spatial or temporal differences between routine and high-risk activities even in different lighting conditions, motion blur, and environmental noise. As observed in Figure 16, it is evident that the trends of the ROC curves of the three classes are clustered in the upper-left part of the plot, indicating the high sensitivity (true positive rate) with the few false alarms. This finding empirically confirms that the meta-level fusion in DeReV is able to combine a variety of visual representations trained on its base models (ResNet, DenseNet, and VGG variants), yielding a well-generalized and stable classifier. In its turn, these results support the appropriateness of the suggested system in the real-time safety observation in urban areas.

**Figure 17:**
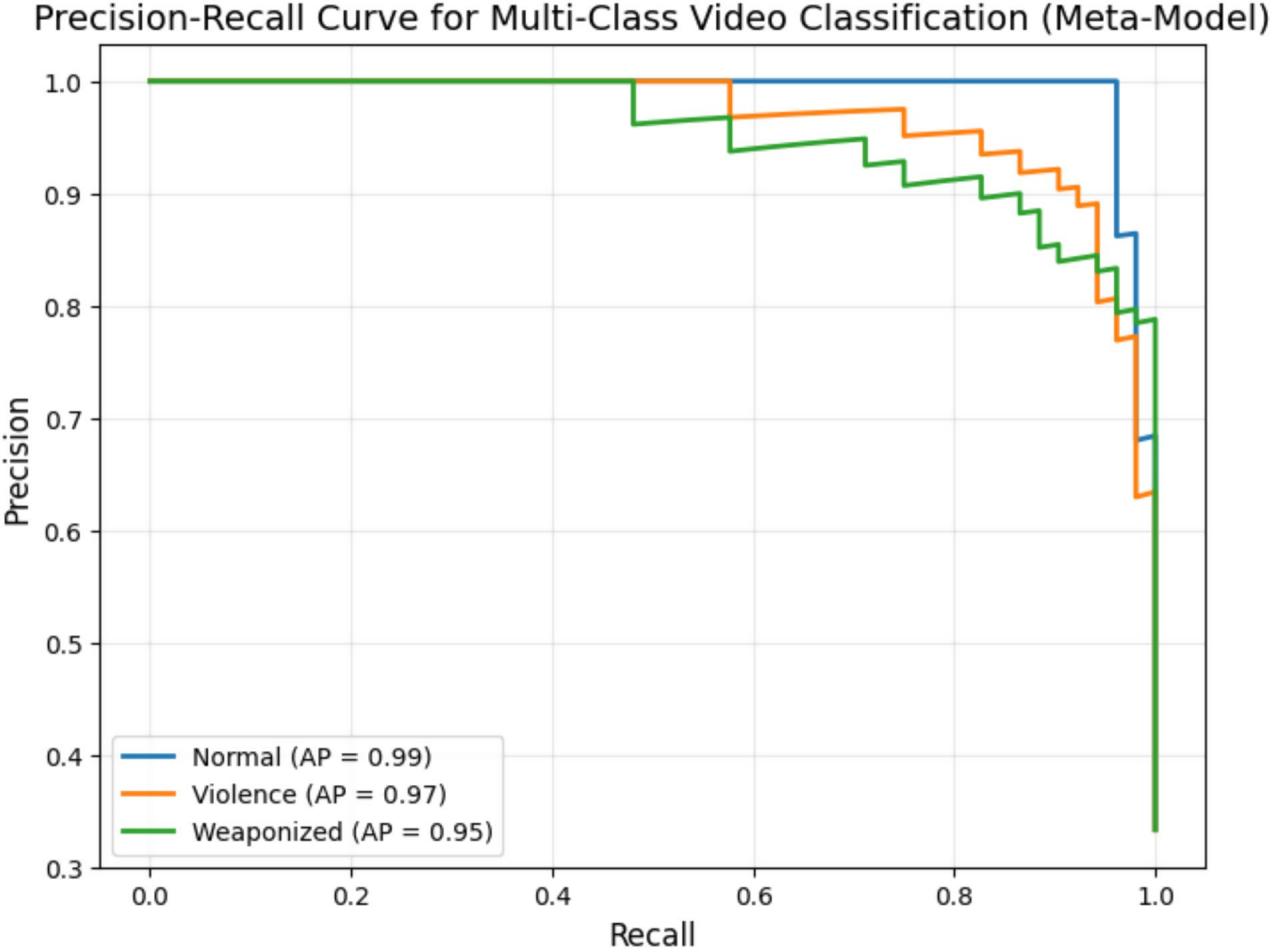
PR curve for DeReV.

To supplement the ROC analysis and obtain a more detailed analysis of the performance in classes, a Precision-Recall (PR) analysis was done on the proposed DeReV architecture. ROC curves are useful when evaluating overall classification performance, but PR curves can be especially informative when the data is imbalanced or the cost of false positives and false negatives is asymmetric, such as those commonly seen in the surveillance systems in practice. The PR curve is a trade-off between precision (positive predictive value) and recall (sensitivity) at different thresholds that provides a narrower view of the detection reliability of the model, particularly when dealing with activities that are rare or weaponized. The DeReV model as shown in Figure 17 has strong precision-recall behavior with all behavioral classes, indicating that it is a very robust model of behavior in terms of its ability to achieve high detection rates as well as not too many false alarms. The model quantitatively scored high in terms of Average Precision (AP) of 0.99 in the case of Normal, 0.97 in the case of Violence, and 0.95 in the case of Weaponized categories. The values show that the model finds true positive cases every time and eliminates false positives as much as possible even in difficult or aesthetically qualitative scenes. Highly evident in Figure 17 is the fact that precision-recall curves of all classes follow a steep and constant profile, which is yet another confirmation of the strength of generalization and depth of discriminatory nature of the ensemble architecture. The fact that AP of Weaponized is slightly lower due to the inherent difficulty of differentiating the subtle weapon cues from the similar non-threatening objects; nevertheless, the performance is extremely high.

### 5.2 Explainability

**Figure 18:**
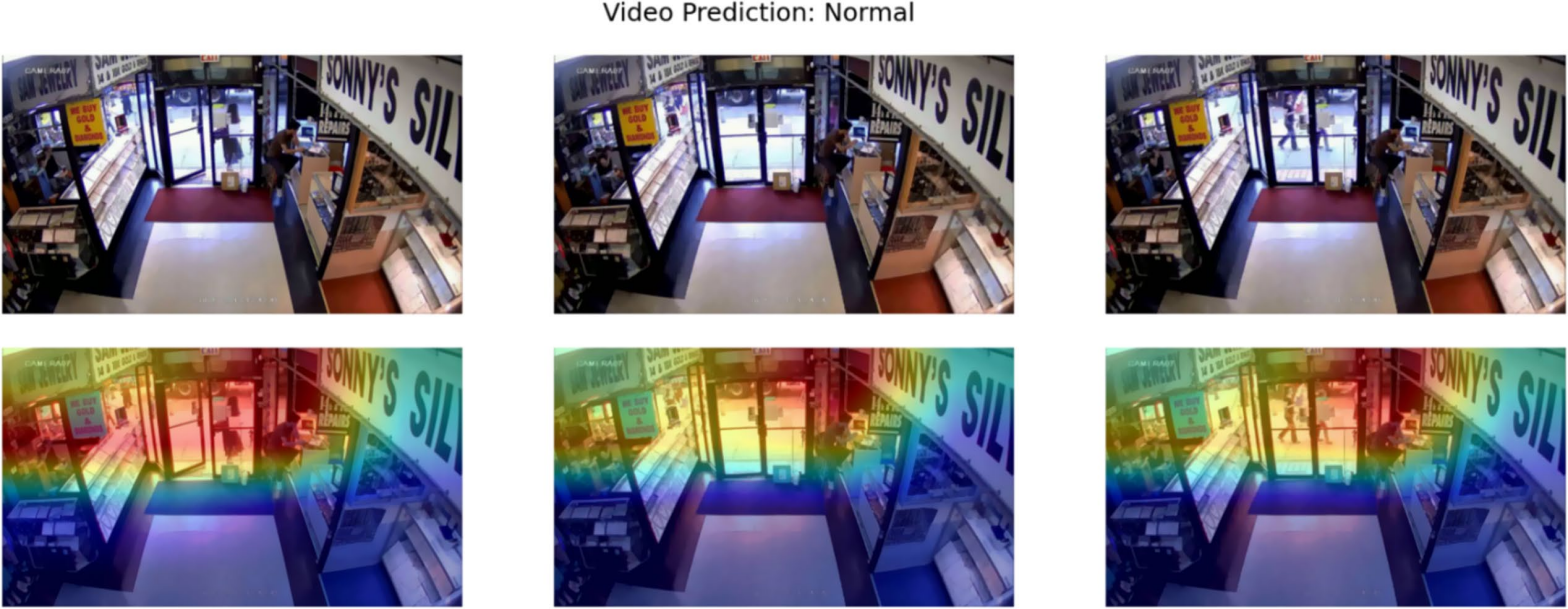
Grad-CAM visualization.

To demonstrate the explainability and visual interpretability of the decision-making process of the proposed DeReV feature-fusion framework, Gradient-weighted Class Activation Mapping (Grad-CAM) was applied to representative frames extracted from test video sequences. Grad-CAM enables visualization of the most discriminative spatial regions influencing the model’s prediction by utilizing the gradients of the target class score with respect to the feature maps of the final convolutional layer. One of the main areas where this visualization technique can be useful is in surveillance uses, as it is used to indicate whether the model is concentrating on contextually significant features, like human interactions, weapon-like objects, or violent body posture in the use of complex urban environments. In this analysis, one hundred and sixty frames of each of the chosen test video sequences were sampled. Based on them, three representative frames were selected to create Grad-CAM heatmaps and analyze the temporal consistency of attention patterns of the model. Figure 18 shows the obtained heatmaps, which clearly show which areas of the space made the biggest contribution to the final classification.

The model is comparatively diffusive in the focus in both the normal activity sequences, and moderate focus on human silhouettes and overview of the scene is indicative of non-aggressive behavior and non-changing environmental interaction. Conversely, in violence and weaponized sequences, the Grad-CAM images have focal regions of activation, which are related to aggressive movement sites, body contacts, and weapons. This set of focused activation processes suggests that the model takes advantage of semantically meaningful characteristics of detecting abnormal or threat-related behaviors.

**Figure 19:**
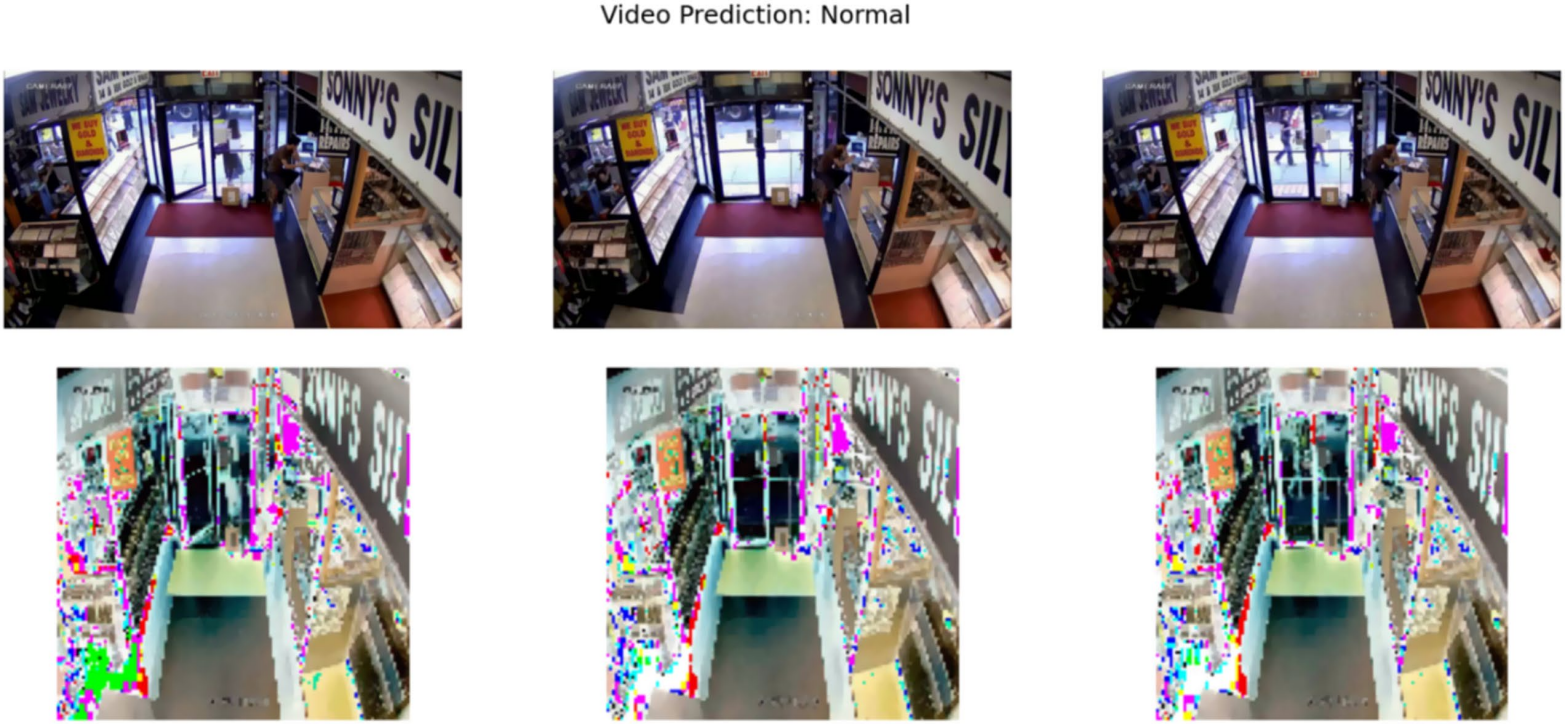
LIME-based interpretability visualization.

In order to make the proposed DeReV feature-fusion framework more interpretable, the Local Interpretable Model-Agnostic Explanations (LIME) method was inspired by some video frames. LIME produces visual descriptions by modifying parts of the original image and observing variations in the output probabilities of the model and thus determines superpixel regions that drive the most noticeable change in the output prediction. This experiment has used test videos that were uniformly sampled and then resized to 128 × 128 pixels before being processed by the trained DeReV model to infer the test video. In every frame picked, the LIME explainer was used to discover the spatial regions that make positive contributions to the predicted label. The discriminative cues that were localized highlighted in the superpixels include human gestures, shapes of motion patterns, interaction zones, and the shape of objects. The resulting visualizations suggest that the model separates the differences between the behaviors of normality, violence, and weaponized by emphasizing more on contextually relevant space areas and ignoring meaningless background textures. Specifically, the points of focus were linked with body posture, interactions in a group, violent gestures, and the availability of possible weapons. These findings contribute to the interpretability of the given framework and ensure the fact that the learned representations coincide with the semantically relevant features. An example of the visualizations of LIME is shown in Figure 19, where the salient superpixel areas that have the strongest contribution in the decision made by the model are highlighted. The figure descriptions give an understanding of the space reasoning behind video-based violence detection and add to the transparency needed to make citizen-centric smart city surveillance applications.

**Figure 20:**
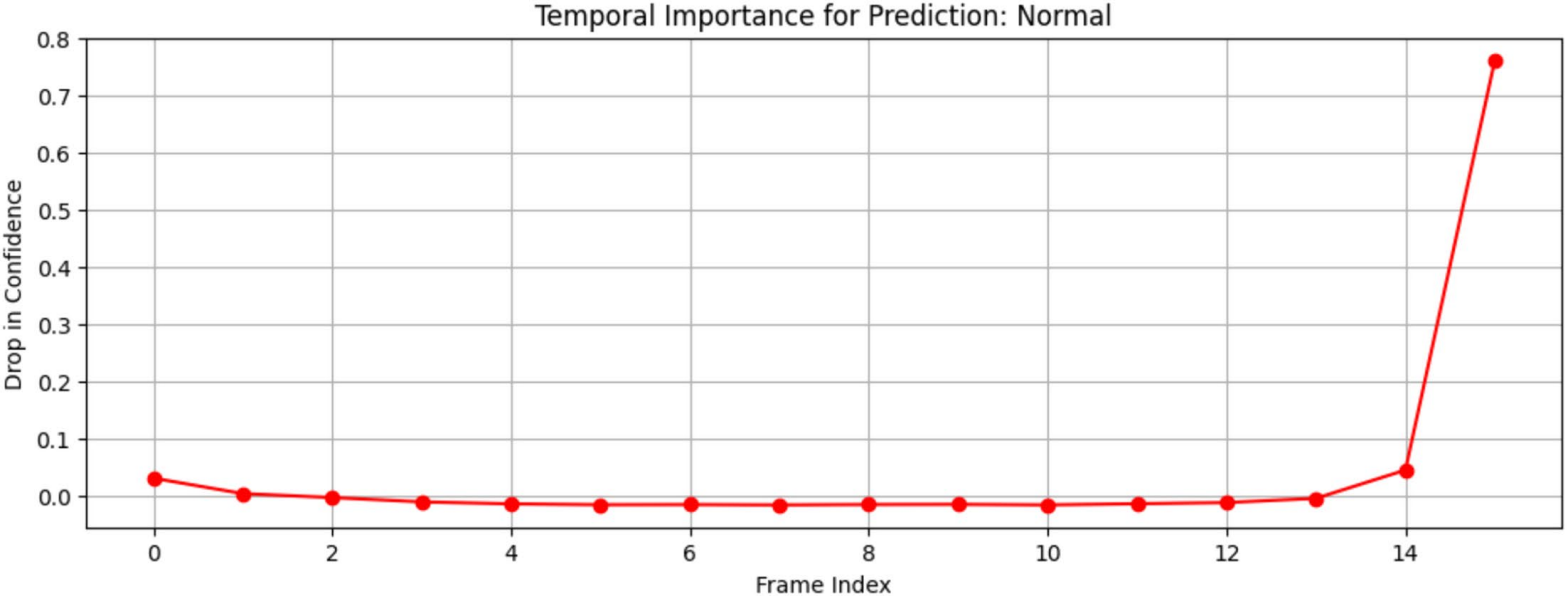
Temporal importance for prediction.

**Figure 21:**
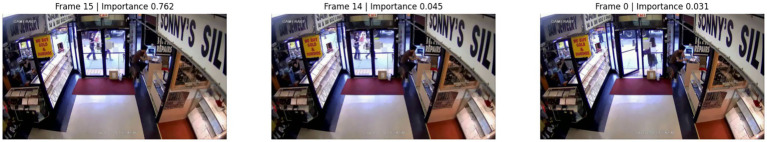
Top three important frames (counterfactual).

To explore the topic of temporal reasoning in the DeReV framework further, the counterfactual temporal importance analysis was carried out. The method assesses individual frame contribution to the final prediction, sequentially masking every frame and re-classifying the frame. The probability of prediction was recalculated without one frame in each video. Frames that led to the greatest reduction in confidence in predictions were labeled as the most significant ones in the decision-making process. This step measures the temporal significance of every frame and demonstrates the role of motion dynamics in the classification results. The temporal importance curve (Figure 20) shows the variability of the confidence in the 16-frame sequence in which high peaks are attributed to the frame with the most influence on the overall predicted class. These peaks are usually associated with major behavioral changes like sudden movement, aggressive contact, or the emergence of a weapon-like item. The three most powerful frames as determined during the counterfactual analysis are pointed out in Figure 21. These frames represent those instances that give the maximum decrease in confidence upon deletion, and therefore, they are critical to the end classification. The interpretability of the results in time illustrated that the DeReV framework is effective in terms of modeling sequential dependencies and also uses motion-based cues in addition to the use of spatial features. The explainability analysis primarily relies on qualitative visual explanations using Grad-CAM, LIME, and temporal counterfactuals to informally understand the decision-making process. These approaches point to semantically relevant spatial and temporal regions involved in violent and weaponized behaviors.

The experiment results reinforce the effectiveness and efficiency of the proposed DeReV framework in smart city surveillance systems. Even more, there is strong evidence that the proposed DeReV framework is successful and efficient. Compared to all the other evaluations, including accuracy in classification, ROC, and precision-recall analysis, frame-level interpretability (Grad-CAM and LIME) and temporal importance measurement, the model was discovered to be more capable of discovering discriminative spatiotemporal characteristics and maintaining high confidence in its decision-making. The near-perfect score of the AUC and mean accuracy of all three groups (normal, violence, and weaponized) prove the fact that the suggested framework is efficient in distinguishing between subtle motion and object shapes even in the highly complicated urban environment. Also, the explainability results suggest that attention in the model is associated with semantically informative regions and temporally significant frames, which show that it possesses reasoning abilities in space as well as time. The experimental assessments of the proposed framework were carried out on the SCVD dataset, which offers realistic surveillance scenes with various activity classifications. In addition to quantitative evaluation to gain insight into the performance, the proposed framework was also tested qualitatively, using representative frames from video clips. Multiclass visualization experiments were conducted using representative test videos from the SCVD dataset, where equally spaced frames were considered to capture the spatial and temporal aspects of various activities. This allows us to assess the robust performance of the proposed framework across different motion, object, and scene characteristics in realistic city scenes. The cloud–edge paradigm is introduced as a deployment platform to enable scalable implementation of the proposed framework. Lightweight preprocessing steps like frame extraction and normalization may be performed on edge devices, and complex processing such as multi-backbone feature extraction and classification are carried out at the cloud level. This approach allows for effective load balancing and scalability in surveillance applications. The findings confirm the fact that DeReV is an effective system that combines deep residual learning and multi-backbone feature fusion to deliver reliable, interpretable, and contextual video understanding to answer the main question of reliability and trustworthiness of next-generation smart surveillance architecture.

## 6 Conclusion

This paper proposed an explainable deep feature-fusion model, DeReV, comprising DenseNet, ResNet, and VGG architectures integrated through a unified feature-level fusion design to achieve better multi-class violence detection in intelligent surveillance systems. The model was tested on the SCVD dataset, which has real-world CCTV videos that are categorized as Normal, Violence, and Weaponized. The experimental results prove that the proposed DeReV framework was much more successful than all the single models of the baseline. DeReV, as depicted by the classification result, had the best accuracy of 96.15 and the macro and weighted average F1-score of 0.9617, which implies that it showed consistency in all the classes. The framework scored the precision at 0.9634, recall at 0.9615, and especially high scores of per-class F1-scores of 0.9903 (normal), 0.9505 (violence), and 0.9444 (weaponized). These values indicate the high potential of the framework to properly detect both violent and weaponized situations with fewer false positives. By contrast, the highest-performing baseline models of VGG16 and DenseNet169 had an accuracy of 92.95 and 92.31%, respectively, and ResNet101 achieved 91.67, and ResNet50 achieved 90.38. Even though these architectures showed good individual performance, their performances were always worse than the DeReV feature-fusion framework, which confirmed the significance of multi-backbone feature integration in terms of enhanced representation learning and better generalization. Additional ROC (Receiver Operating Characteristic) and Precision-Recall (PR) curve analysis helped to give a better understanding of the reliability and discriminative strength of the DeReV framework. The ROC analysis has an AUC of 0.99 using the Normal class, 0.98 using the Violence class, and 0.98 using the Weaponized class, which shows that the analysis is very accurate in class separation and generalization. Similarly, the analysis of the PR curve provided 0.99, 0.97, and 0.95 values of AUC in the case of Normal, Violence, and Weaponized, indicating a properly preserved balance between precision and recall at different thresholds. These findings verify the strength and soundness of the proposed model to identify various safety situations in an urban environment with high integrity. The incorporation of XAI models, namely Grad-CAM, LIME, and Temporal Counterfactual Analysis, offered interpretability into the process of making decisions by the model. Grad-CAM visualizations identified spatial locations in frames having predictive power, LIME provided local interpretability of predictions, and temporal counterfactual analysis could identify the most significant (usually non-uniform) frames, which contributed the most to decision confidence.

In the future, the proposed framework will be extended by integrating audio signals and IoT sensor feeds as multi-modal data sources to enhance situational awareness and reduce false detections. Real-time edge deployment and optimization strategies will be explored to enable low-latency violence detection suitable for large-scale smart city infrastructures. The proposed framework will also be equipped with the detailed benchmarking of inference latency, memory usage, and energy efficiency across different hardware configurations to further validate real-time deployment feasibility. Furthermore, analyzing cross-dataset generalization and incorporating federated learning strategies can improve model adaptability, privacy preservation, and resilience across diverse urban environments.

## Data Availability

Publicly available datasets were analyzed in this study. This data can be found at: https://www.kaggle.com/datasets/toluwaniaremu/smartcity-cctv-violence-detection-dataset-scvd.
